# The association between iron status and some immunological factors in the pregnancy

**Published:** 2011

**Authors:** Seyed Alireza Sobhani, Zahra Etaati, Sepideh Mirani, Paknoosh Saberi, Mahnaz Shiroodi, Hojjat Salmasian, Nadereh Naderi

**Affiliations:** 1 Department of Pathology, Hormozgan University of Medical Sciences, Bandar Abbas, Iran.; 2 Department of Obstetrics and Gynecology, Hormozgan University of Medical Sciences, Bandar Abbas, Iran.; 3 General physician, Hormozgan University of Medical Sciences, Bandar Abbas, Iran.; 4 Department of Biochemistry, Hormozgan University of Medical Sciences, Bandar Abbas, Iran.; 5 Center for research of Molecular Medicine, Hormozgan University of Medical Sciences, Bandar Abbas, Iran.; 6 Research Fellow, Tehran University of Medical Sciences, Tehran, Iran.; 7 Research Fellow, Farzan Clinical Research Institute, Tehran, Iran.; 8 Department of Immunology, Hormozgan University of Medical Science, Bandar Abbas, Iran.

Iron deficiency anemia (IDA) is a common problem in many developing countries. It is still considered the most common nutrition deficiency worldwide. Apart from its direct hematologic importance, IDA affects cellular and humoral immunity and predisposes the host to infections (1).

Pregnant women are highly prone to IDA. Controversial results are reported in studies targeting this group of patients. Tang *et al* showed a direct association between hemoglobin concentration and the count of CD4^+^ T-cell lymphocytes, serum levels of IL-2 and IgG, and an inverse association with susceptibility to infection (2). Ironically, Leush *et al* reported an increase in IgM and IgG in the second and third trimesters of pregnancy in women with IDA (3).

With regard to controversial results and the scarcity of studies focusing on pregnant women, we aimed to enlighten the relation between iron status and some immunological factors include some component of complement system, IgA, IgM, IgG subclasses of immunoglobulins and pro-inflammatory cytokines during the third trimester of pregnancy. 

In a descriptive-analytic study participants were recruited using convenient sampling from the women in the third trimester of pregnancy referred to the labor room of gynecology and obstetrics ward of Dr. Shariati Hospital of Bandar Abbas, Iran. Patients with signs and symptoms of thalassemia, infectious diseases or autoimmune diseases were excluded.

IDA were defined with two criteria, hemoglobin concentration of less than 10 mg/dL (its normal range during the third trimesters of pregnancy is 11-14mg/dL) (4) and ferritin less than 40 ng/dL. Patients were categorized into two groups: those with iron deficiency anemia (IDA) and those without this condition (no IDA). 

Red cell indices including hemoglobin (Hb) levels, hematocrit (HCT), mean corpuscular volume (MCV), red blood cell distribution width (RDW), and mean corpuscular hemoglobin (MCH), serum iron (SI) and total iron binding capacity (TIBC), concentration of ferritin, C3 and C4 complements and IgA, IgM and IgG subclasses of immunoglobulins were determined. Data was analyzed using SPSS version 11.5 using Student t-test, Pearson’s correlation test and Kolmogorov-Smirnov’s test of normal distribution. 

Ninety-two patients were studied. They were aged between 15 and 42 years (mean=25.69±6.2). According to our definition of IDA in pregnancy, 21 patients (22.8%) had IDA. 

Our analysis of differences between the two groups in regard to immunologic markers showed that C_4_ levels are lower in the IDA group (p=0.009) and the levels of C_3_, IgM, IgG, IgA, IL-1, IL-6 and TNF-α were not statistically different in the two groups .

We noticed that higher levels of serum iron are correlated with higher levels of C_3_, C_4_ and IgG_1_. Due to important properties of IgG1 like complement fixation and opsonic activity, this subclass is dominant antibody to pneumococcal capsular polysaccharides and its deficiency is associated with current infections (5). Taken together noticing key roles of C3, C4 in complement-mediated bacteriolysis, opsonization, facilitated ingestion immune adherence (6) and association of C3, C4 with Iron serum levels found in this study we suggest that decreased level of Iron increases susceptibility of pregnant women to infections like chronic bacterial respiratory infections and recurrent genital herpes (5). Analyzing immunologic parameters differences between the two groups of IDA and no IDA we found that C4 levels are lower in the IDA group but not the levels of C3, IgM, IgG, IgA, IL-1, IL-6 and TNF-α .Our findings about IgM, IgG, IgA are in contradiction to scanty studies in this field. Tang *et al* (2) about significantly lower level of IgG, CD3+ and CD4+ cells, the ratio of CD4+/CD8+cells, serum IL-2 in second trimester of IDA pregnant woman and Leush *et al* (3) study showed increase of IgM and IgG in second and third trimesters of IDA groups. 

Our findings about non-significant difference in C3 and significant difference in C4 levels in IDA and no IDA groups is in agreement with Galan *et al* report about significantly positive correlation of C4 IgA, IgM and Serum ferritin (7). Despite of mounting evidence that TNF, IL-1, and IL-6 cytokines affect hemopoiesis and iron metabolism there was no significant association between IL-1, IL-6 and TNF-α and serum Iron, ferritin, TIBC in our study and inflammatory cytokines were statistically indifferent in the two IDA and no IDA groups. To our knowledge, there are no reports on inflammatory cytokine levels and Iron parameters in pregnant women, but a limited number of reports exploring this field in children and adults. 

Bergman *et al* which analyzed the in vitro production of IL-1beta, IL-2, IL-6, IL-10, and TNF-α by peripheral blood mononuclear cells from 20 patients with IDA report that the secretion of the cytokines other than IL-2 did not differ from that of controls (8). In another study there was no difference in serum levels of IL-6 in iron deficiency anemia before and after iron supplementation in children with IDA but in the iron-deficiency group the production of IL-2 was found to be significantly lower than that in controls and became normal after iron supplementation (9). Safuanova *et al* work about adequate therapy by iron-containing drugs in IDA patients resulted in decreased concentrations of IL-1, IL-6, TNF-alpha and INF-gamma and recovering the functional status of the immune system (10). It is possible that discrepancy seen between our results and mentioned reports in non-pregnant patients is a reflection of dramatic change of immune function in pregnancy. 

Analyze of the results of our study and similar researches leads us to the conclusion that unlike extensive immunological changes have been observed in children, IDA has little effect on humoral immunity system of pregnant women, but decrease in serum iron could predispose them to pyogenic infection and may predict increased susceptibility IDA pregnant women to infections. 
